# Characterization of acute TLR-7 agonist-induced hemorrhagic myocarditis in mice by multiparametric quantitative cardiac magnetic resonance imaging

**DOI:** 10.1242/dmm.040725

**Published:** 2019-08-16

**Authors:** Nicoleta Baxan, Angelos Papanikolaou, Isabelle Salles-Crawley, Amrit Lota, Rasheda Chowdhury, Olivier Dubois, Jane Branca, Muneer G. Hasham, Nadia Rosenthal, Sanjay K. Prasad, Lan Zhao, Sian E. Harding, Susanne Sattler

**Affiliations:** 1 Biological Imaging Centre, Department of Medicine, Imperial College London, London W12 0NN, UK; 2 National Heart and Lung Institute, Imperial College London, London W12 0NN, UK; 3 Royal Brompton Hospital, Royal Brompton and Harefield NHS Foundation Trust, London SW3 6NP, UK; 4 The Jackson Laboratory, 600 Main Street, Bar Harbor, ME 04609, USA

**Keywords:** Cardiac hemorrhage, Myocarditis, Resiquimod, TLR-7, Cardiac magnetic resonance imaging, CMR, MRI

## Abstract

Hemorrhagic myocarditis is a potentially fatal complication of excessive levels of systemic inflammation. It has been reported in viral infection, but is also possible in systemic autoimmunity. Epicutaneous treatment of mice with the Toll-like receptor 7 (TLR-7) agonist Resiquimod induces auto-antibodies and systemic tissue damage, including in the heart, and is used as an inducible mouse model of systemic lupus erythematosus (SLE). Here, we show that overactivation of the TLR-7 pathway of viral recognition by Resiquimod treatment of CFN mice induces severe thrombocytopenia and internal bleeding, which manifests most prominently as hemorrhagic myocarditis. We optimized a cardiac magnetic resonance (CMR) tissue mapping approach for the *in vivo* detection of diffuse infiltration, fibrosis and hemorrhages using a combination of T_1_, T_2_ and T_2_^*^ relaxation times, and compared results with *ex vivo* histopathology of cardiac sections corresponding to CMR tissue maps. This allowed detailed correlation between *in vivo* CMR parameters and *ex vivo* histopathology, and confirmed the need to include T_2_^*^ measurements to detect tissue iron for accurate interpretation of pathology associated with CMR parameter changes. In summary, we provide detailed histological and *in vivo* imaging-based characterization of acute hemorrhagic myocarditis as an acute cardiac complication in the mouse model of Resiquimod-induced SLE, and a refined CMR protocol to allow non-invasive longitudinal *in vivo* studies of heart involvement in acute inflammation. We propose that adding T_2_^*^ mapping to CMR protocols for myocarditis diagnosis improves diagnostic sensitivity and interpretation of disease mechanisms.

This article has an associated First Person interview with the first author of the paper.

## Abstract (precis)

**Summary:** The authors present a mouse model of acute hemorrhagic myocarditis and a refined technique of preclinical cardiac MR imaging to allow non-invasive detection of diffuse myocardial tissue damage accounting for iron deposition.

## INTRODUCTION

Immune-mediated damage to the heart can occur as the result of a wide variety of underlying conditions such as infectious disease, exposure to toxins, chemotherapeutic agents, immune checkpoint inhibitors and systemic inflammation caused by autoimmune disease. Besides being caused by a range of viruses known to induce severe hemorrhagic fevers, viral myocarditis can also be a complication of more common viruses, including coxsackie virus (Freund et al., 2010), adenovirus (Casas et al., 2005) and influenza (Ukimura et al., 2012). In rare cases, viral infection can trigger excessive systemic inflammation and bleeding, which may manifest as hemorrhages in internal tissues and organs, including the heart (Gdynia et al., 2011; Edler et al., 2011; Tenenbaum et al., 2005). Importantly, because of the severity of other clinical symptoms, myocarditis can remain undetected and diagnosis often only occurs post mortem (Treacy et al., 2010). Acute hemorrhagic myocarditis has also been reported in systemic lupus erythematosus (SLE) (Dickens et al., 1992). In addition, despite clearance of the initial viral infection, inflammation may persist because of the development of self-directed immune responses leading to persistent inflammatory cardiomyopathy characterized by myocardial contractile dysfunction similar to dilated cardiomyopathy (Caforio et al., 2017). Although clinically detected cardiac hemorrhage is rare (Dickens et al., 1992), it is feasible that inflammatory effects on hematological parameters and vasculature cause subclinical extravasation of red blood cells, which may result in tissue iron deposition.

Despite poor pathological specificity, cardiac magnetic resonance (CMR) imaging is a useful tool for non-invasive *in vivo* tissue characterization under highly representative physiological conditions and is clinically used to detect tissue damage in inflammatory disease (Mavrogeni et al., 2017; Ferreira et al., 2018; Anderson et al., 2001). However, potential iron deposition needs to be considered because the paramagnetic properties of iron strongly influence CMR relaxation times.

Although various cardiac structures have been reported to be affected in mouse models of acute systemic inflammation (Sanghera et al., 2019), the specific phenotype of hemorrhagic myocarditis under such conditions has only been detected in the acute phase of the SLE model induced by application of the Toll-like receptor 7 (TLR-7) agonist Resiquimod (Yokogawa et al., 2014; Hasham et al., 2017).

Here, we further characterize the acute response of CFN mice, a previously described recombinant inbred mouse line obtained by crossing C57BL/6J, FVB/NJ and NOD/ShiLtJ parental lines (Hasham et al., 2017), to Resiquimod treatment. We provide a thorough side-by-side comparison of the *ex vivo* histopathology of the heart and CMR parameters to establish a protocol for contrast agent-free CMR parametric mapping for the non-invasive *in vivo* detection of diffuse immune-mediated damage, taking into account the potential presence of hemorrhage.

## RESULTS

### TLR-7 agonist Resiquimod induces severe pan-cardiac hemorrhage and inflammatory tissue damage

**Fig. 1.**
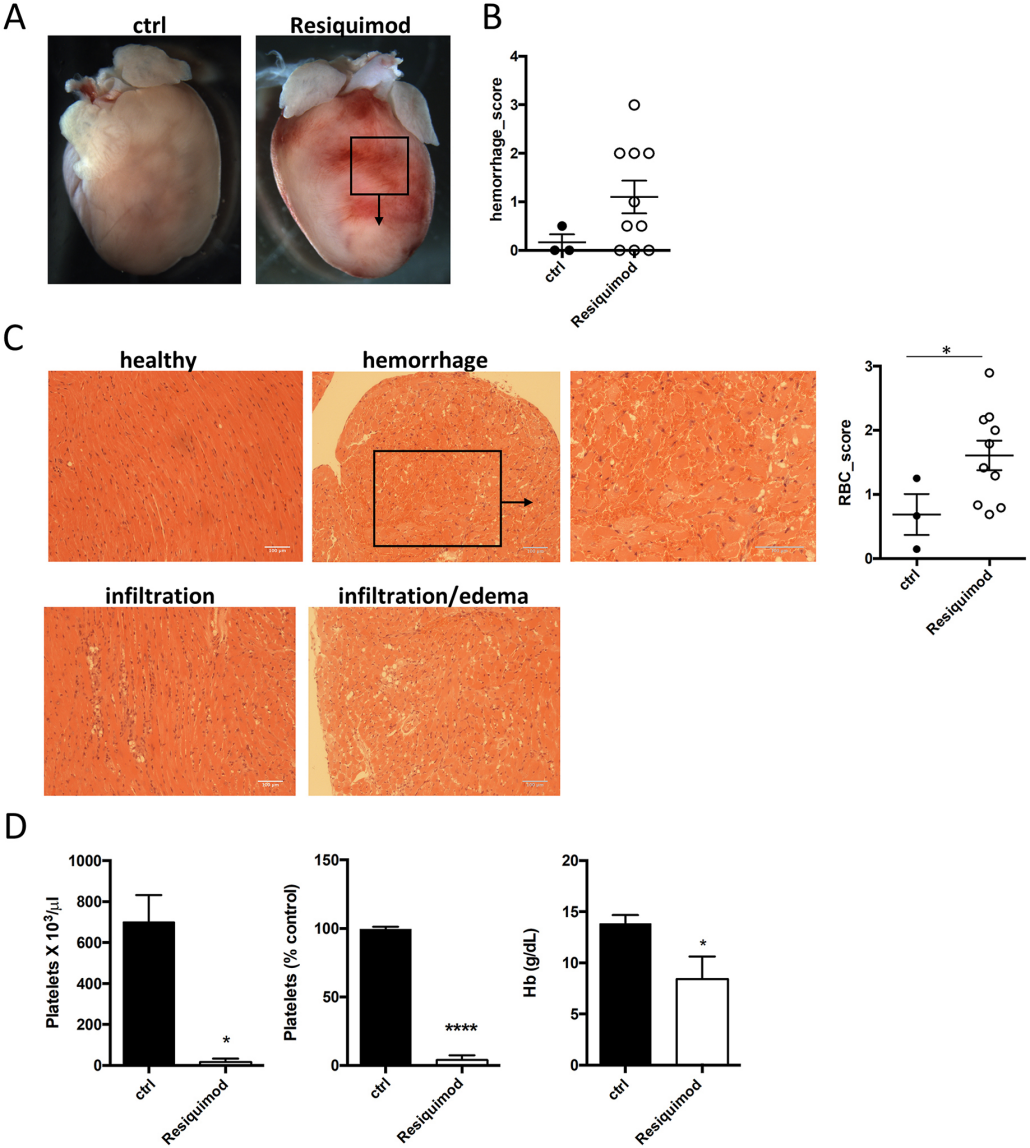
**TLR-7 agonist Resiquimod induces myocarditis, thrombocytopenia and cardiac hemorrhage.** Cardiac involvement in Resiquimod-treated mice was assessed macroscopically and by microscopic histopathology. (A,B) Example and quantification of macroscopic hemorrhagic lesions on hearts of Resiquimod-treated mice. (C) Representative micrographs of H&E-stained heart sections of Resiquimod-treated mice showing immune cell infiltration, edema and red blood cells in the myocardial interstitial space. Degree of RBC extravasation was scored on a scale from 0 to 3, where 0 indicates none and 3 is the most severe. (D) Full platelet count, percentage drop in platelet number from baseline, and changes in hemoglobin content in response to Resiquimod-treatment. Mann–Whitney test was used for semiquantitative scores, *n*=3 (ctrl), *n*=10 (treated); unpaired two-tailed Student's *t*-test for hematological analysis, *n*=3. Symbols represent individual animals. All data presented as mean±s.e.m. *P<0.05, ****P<0.001.

As also shown previously (Hasham et al., 2017), Resiquimod treatment induced patches of cardiac hemorrhage that were severe enough to be macroscopically visible on the surface of the heart in the majority of treated mice (Fig. 1A,B). Histopathological examination revealed immune cell infiltration, edema, cardiomyocyte damage and accumulation of red blood cells (RBC) between myocardial fibers (Fig. 1C). RBC extravasation into the myocardium was evident (Fig. 1C, graph). Resiquimod-treated mice also developed clinical hematological manifestations, including severe thrombocytopenia and anemia (Fig. 1D) after only two weeks of treatment.

### Native CMR T_1_ and T_2_ tissue mapping indicates inflammatory damage, but regional values do not conclusively correlate with histopathology

**Fig. 2.**
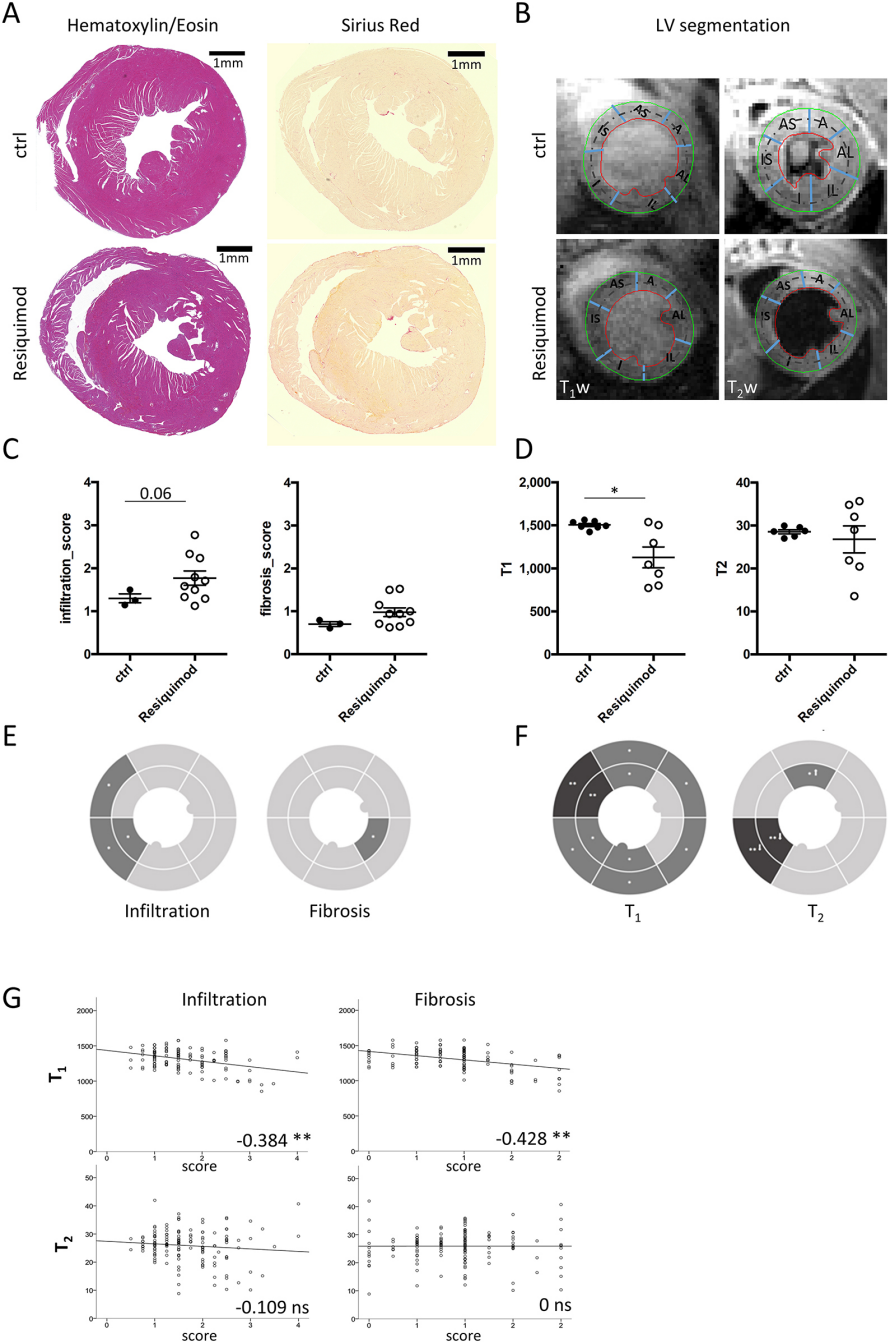
**Native T_1_ and T_2_ tissue mapping does not conclusively detect inflammatory cardiac damage in Resiquimod-treated mice.** Heart tissue damage in Resiquimod-treated mice was assessed by histology and MRI. (A) Example of H&E and Picrosirius Red-stained heart sections showing immune cell infiltration and fibrosis. (B) Example of corresponding T_1_w and T_2_w images to demonstrate segmentation (A, anterior; AL, anterior lateral; AS, anterior septum; I, inferior; IL, inferior lateral; IS, inferior septum; T_1_w, T_1_ weighted; T_2_w, T_2_ weighted). (C) Global semiquantitative score for infiltration, fibrosis and RBC extravasation on a scale from 0 to 3, where 0 indicates none and 3 is the most severe. (D) Global values for T_1_ and T_2_ values of Resiquimod-treated mice compared with healthy controls. The Mann–Whitney test was used for semiquantitative histopathology scores, *n*=3 (ctrl), *n*=10 (treated); paired Student's two-tailed *t*-test for longitudinal MRI values, *n*=7; *P<0.05. (E,F) Regional analysis depicted as pie charts performed on 12 segments at mid level per mouse, showing infiltration, fibrosis and RBC accumulation (E), and T_1_ and T_2_ (F). Shading indicates significance compared with controls; *P<0.05, **P<0.005. Arrows indicate increase (↑) or decrease (↓) of values compared with controls. (G) Correlation between T_1_ and T_2_ indices with individual histopathology scores. Pearson's correlation was used, pooling data from three independent experiments, *n*=10-12 (ctrl), *n*=9-10 (treated); *correlation significant at the 0.01 level, **correlation significant at the 0.05 level. Symbols represent individual animals; all data presented as mean±s.e.m.

Resiquimod-induced systemic inflammation induced myocarditis, as assessed by immune cell infiltration in histological sections (Fig. 2A). Global CMR T_1_ and T_2_ relaxation times were extracted from the mid slice of the heart for comparison with histopathological changes in corresponding histology sections (Fig. 2B). Global histopathological damage scores showed mild changes compared with baseline values for inflammatory infiltration in Resiquimod-treated mice compared with untreated animals [*n*=3 (control), *n*=10 (treated)]. Collagen deposition as a measure of fibrosis was negligible, which is likely owing to the early acute stage of disease (Fig. 2C). Global T_1_ values decreased significantly (1128±121 ms, *P*=0.017) compared with values from myocardial tissue of untreated mice (1505±18.5 ms), whereas T_2_ did not change (28.5±0.5 versus 27±3 ms); *n*=7 (control and treated) (Fig. 2D).

Regional analysis performed on 12 heart segments (Fig. 2E) revealed that the intraventricular septum and subepicardial areas were most severely affected, whereas left ventricular (LV) free wall and subendocardium showed only mild inflammatory damage. In line with this, CMR imaging detected a significant drop in subepicardial T_1_ values, particularly in the anterior part of the intraventricular septum. Regional T_2_ values remained largely unchanged, except for the inferior septum (Fig. 2F) where values dropped significantly. Despite a seemingly similar pattern of spatial distribution between changes in regional T_1_ and T_2_ values and the corresponding histopathological scores for infiltration (Fig. 2E,F), correlation as tested by pairwise Pearson's test [*n*=10-12 (control), *n*=9-10 (treated)*] was only mild for T_1_ (infiltration −0.384, fibrosis −0.428) and absent for T_2_ (infiltration −0.109, fibrosis 0) (Fig. 2G). Specific regions seemed to contradict expectations of increased T_1_ and T_2_ values based on histopathology. For example, native T_1_ values decreased significantly in the intraventricular septum and along the entire subepicardium, which was in stark contrast to the anticipated increase resulting from histologically observed immune cell infiltration (Fig. 2E,F).

### Tissue iron deposits influence regional T_1_ and T_2_ measurements

**Fig. 3.**
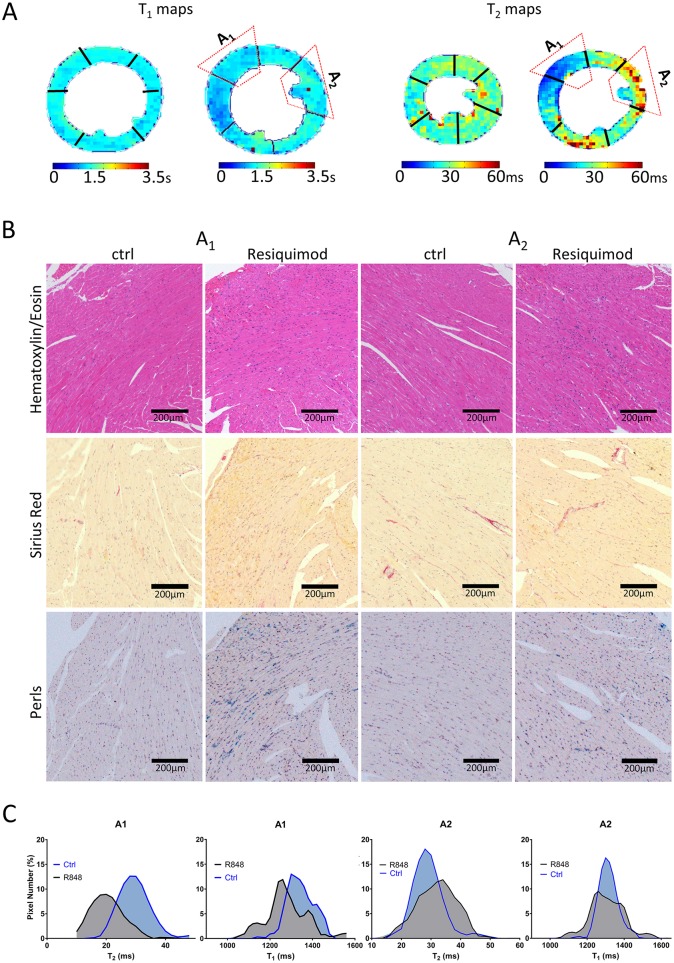
**Regional T_1_ and T_2_ mapping is strongly influenced by iron deposition in the tissue.** Heart tissue damage was assessed by histology and MRI. (A) T_1_ and T_2_ maps illustrating heterogeneity of T_1_ and T_2_ relaxation times with regions of low T_1_, T_2_ (A_1_) and high T_2_ (A_2_). (B) Corresponding areas of interest (A_1_ and A_2_) in paraffin-embedded heart sections stained with H&E, Picrosirius Red and Perls Prussian Blue. (C) Histograms of signal distribution of T_1_ and T_2_ indices from regions of interest A_1_ and A_2_.

Although we did identify areas of increased T_2_ values corresponding to areas of inflammatory infiltration and edema in histology, as expected (region of interest A_2_, Fig. 3A) other areas with comparable levels of immune cell infiltration yielded unchanged or even decreased T_2_ values (region of interest A_1_, Fig. 3A). Fibrosis was rejected as a likely factor to cause differences in T_2_ values, as levels were negligible and evenly distributed across the heart due to the stage of disease.

Besides inflammation, the second dominant histopathological phenotype in hearts of Resiquimod-treated CFN mice was the substantial accumulation of interstitial erythrocytes, which likely leak into the tissue as a result of impaired hemostasis caused by severe thrombocytopenia and inflammatory damage of the endothelial lining of blood vessels. This erythrocyte accumulation led us to investigate the potential of hemoglobin-derived iron deposition in the heart. Indeed, a significant amount of iron was detected in areas of particularly low T_1_ and T_2_ values (A_1_, Fig. 3B), whereas T_2_ values in areas without iron were decreased in the presence of inflammation, as expected (A_2_, Fig. 3B). Histograms of T_1_ and T_2_ signal distribution were computed from the anterior septum (A_1_, Fig. 3C) and anterior lateral wall (A_2_, Fig. 3C). The signal distribution of T_1_ mapping indices enabled a clear discrimination between regions of healthy myocardium and areas with high iron content (A_1_, Fig. 3C). T_2_ signal distribution behaved similarly, confirming the dominant effect of iron in reducing T_1_ and T_2_ relaxation times. In tissue areas with infiltration, but without iron deposition (A_2_, Fig. 3C), T_1_ showed high accuracy by not picking up the influences of T_2_ relaxation (Puntmann et al., 2013) and was characterized by a more dispersed signal distribution than controls. T_2_ histograms presented a broader signal distribution, with moderate increase in mean values compared with healthy areas, proving the sensitivity of T_2_ mapping in detecting edema and cellular infiltrate only in areas without iron deposition.

In summary, we identified a significant amount of interstitial iron in the hearts of Resiquimod-treated mice, which is most likely the result of hemorrhage, erythrocyte cell death and hemoglobin degradation. The strong paramagnetic characteristics of iron led to a decrease in T_1_ and T_2_ relaxation times. Tissue areas lacking iron deposits followed the classical CMR paradigm that T_1_ detects fibrosis (Puntmann et al., 2013) and T_2_ detects edema and/or cell infiltration (Thavendiranathan et al., 2012).

### A refined T_2_^*^ mapping approach reveals the impact of iron deposition on T_1_ and T_2_ values

Because of its high sensitivity to paramagnetic iron, CMR T_2_^*^ mapping is used to detect and quantify iron storage molecules (ferritin and hemosiderin) deposited in tissue (Langkammer et al., 2010). However, the LV free wall is usually excluded from T_2_^*^ measurements because of large magnetic susceptibility artefacts caused by its proximity to the air-filled lung. Using ultrashort echo time (UTE) instead of the conventional gradient-echo readout (Robson et al., 2003), we obtained artefact-free T_2_^*^ maps with full coverage of the intraventricular septum and LV free wall at apical, mid and basal levels of the mouse heart. Examples of the high-quality T_1_, T_2_ and T_2_^*^ maps acquired in healthy controls are shown in Fig. S1.

**Fig. 4.**
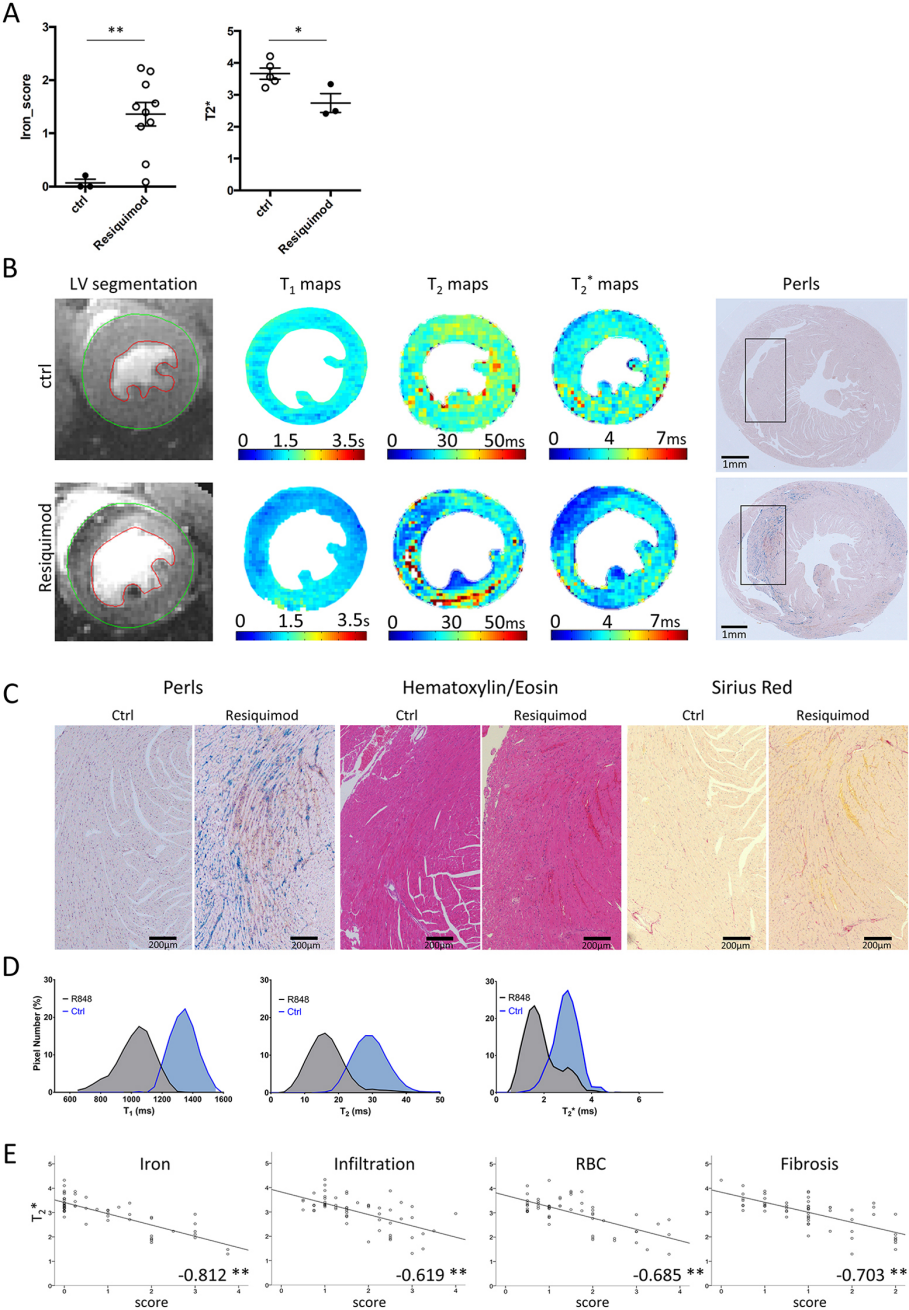
T_2_^*^ reveals severe cardiac iron deposition impacting on T_1_ and T_2_ measurements. Heart tissue damage was assessed by histology and MRI. (A) Global histopathological scores of RBC extravasation, iron and T_2_^*^ values of Resiquimod-treated mice compared with control mice. The Mann–Whitney test was used for semiquantitative histopathology scores, *n*=3 (ctrl), *n*=10 (treated); unpaired two-tailed Student's *t*-test for MRI values, *n*=5 (ctrl), *n*=3 (treated); *P<0.05, **P<0.005. (B) Examples of T_1_, T_2_ and T_2_^*^ maps of mid-section LV with corresponding Perls Prussian Blue-stained paraffin-embedded heart sections of Resiquimod-treated and control mice showing significant iron deposition. (C) High-magnification images of tissue areas with iron deposition (Perls Prussian Blue), immune cell infiltration (H&E) and mild fibrosis (Picrosirius Red). (D) Histograms of T_1_, T_2_ and T_2_^*^ signal distribution in the anterior septum showing the impact of iron on the behavior of each relaxation time index. (E) Correlation between T_2_^*^ values and individual histopathology scores. Pearson's correlation was used, pooling data from three independent experiments, *n*=3-5 (ctrl), *n*=3-10 (treated); *correlation significant at the 0.01 level, **correlation significant at the 0.05 level. Symbols represent individual animals; all data presented as mean±s.e.m.

Erythrocyte extravasation and iron accumulation increased in Resiquimod-treated mice, and global T_2_^*^ values dropped significantly to 3.7±0.2 ms (Fig. 4A). Perls Prussian Blue staining showed a significant increase in tissue iron in myocardial regions with low T_2_ and T_2_^*^ relaxation times. Representative T_1_, T_2_ and T_2_^*^ maps of Resiquimod-treated mice with corresponding histological iron staining of heart sections are presented in Fig. 4B. Histology micrographs were magnified to show an example of severe iron accumulation in the anterior wall of the interventricular septum. The corresponding Picrosirius Red-stained section demonstrated mild interstitial fibrosis and hematoxylin and eosin (H&E) stains showed mononuclear cell infiltration (Fig. 4C). Histograms of T_2_^*^ signal distribution revealed a skewed distribution towards lower median values, consistent with the T_2_^*^ hypo-intense regions of strong iron deposition, whereas the histograms of T_1_ and T_2_ showed low mean values but the normal distribution of signal suggested a weaker sensitivity to iron compared with T_2_^*^ (Fig. 4D).

Correlation analysis between regional T_2_^*^ and histopathological parameters showed strong negative correlations with the degree of iron deposition (−0.812), RBC extravasation (−0.685), infiltration (−0.619) and fibrosis (−0.703) [*n*=3-5 (control), *n*=3-10 (treated)] (Fig. 4E). Most importantly, correlation between T_2_^*^ values and regional iron deposition scores was very strong and highly significant. T_1_ and T_2_ correlations with iron were −0.413 and −0.277, respectively. Correlation between T_2_^*^ and other damage parameters was lower and might be affected by a knock-on effect from their own correlation with iron deposition. Notably, T_1_, T_2_ and T_2_^*^ values did not change in the liver of the same animals (Fig. S2), suggesting that inflammatory damage and hemorrhage in this model are specific to the heart.

## DISCUSSION

Hemorrhagic myocarditis is a potentially fatal complication of acute systemic inflammation. However, to date, a diagnosis is often only obtained post mortem. In addition, acute myocarditis can trigger an autoimmune reaction against the heart (Bracamonte-Baran and Čiháková, 2017) and it is feasible that severity of hemorrhaging, acute tissue damage and subsequent autoimmunity are correlated. In the brain, hemoglobin-derived iron plays a major role in secondary damage following hemorrhage (Stephenson et al., 2014) and iron-overload cardiomyopathy is caused by iron deposition in the tissue, induction of cardiomyocyte cell death and an ensuing inflammatory reaction (Gammella et al., 2015). A recent study of CVB3-induced myocarditis in mice detected iron deposits within infected cardiomyocytes (Helluy et al., 2017), suggesting that, albeit due to a different mechanism, deposition of iron in the heart could be more prominent during pathological inflammatory processes than previously appreciated.

Underlying mechanisms leading to hemorrhage in some myocarditis patients, but not in others, and implications on survival and subsequent development of inflammatory cardiomyopathies are far from understood. This supports the need for thorough mechanistic and therapeutic studies in well-characterized preclinical models. Although various cardiac phenotypes have been reported in mouse models of autoimmunity (Sanghera et al., 2019), the specific phenotype of hemorrhagic myocarditis under such conditions has so far only been studied in Resiquimod-induced systemic inflammation in CFN mice (Hasham et al., 2017). Considering that myocardial hemorrhaging may be more common than currently appreciated in systemic inflammation of both infectious and autoimmune origin, as well as in heart-targeted infections, these processes need to be characterized in the corresponding mouse models. A wide range of such models exists, but they have not yet been analyzed for cardiac hemorrhages. Considering that only a small proportion of human patients develop clinically detected hemorrhagic myocarditis, there is likely a genetic component to disease susceptibility; thus, studying genetically diverse mouse panels instead of a single inbred mouse line could reveal a range of susceptibilities (Graham et al., 2015). Notably, CFN mice were chosen because of their increased sensitivity to Resiquimod treatment compared with the three parental strains C57BL/6J, FVB/NJ and NOD/ShiLtJ (Hasham et al., 2017).

The mouse model used in this study has previously been described at chronic stage as a model of systemic autoimmunity (Yokogawa et al., 2014). Yet, in the early acute phase, it appears to mimic more closely the physiological responses to viral infection, because of stimulation of the TLR-7 pathway. TLR-7 is a pattern recognition receptor involved in recognition of single-stranded RNA of viral origin and is thus crucial in host defense against viral infections (Xagorari and Chlichlia, 2008). It is conceivable that artificial overactivation of this virus defense system causes the same phenomena seen in severe complications of viral infection. This might include disseminated intravascular coagulation (DIC) resulting from acute systemic platelet activation, leading to thrombocytopenia and internal hemorrhage (Goeijenbier et al., 2012). Notably, in an lymphocytic choriomeningitis virus (LCMV)-infection model, it was shown that a severe drop in platelet count of more than 85% is necessary to cause hemorrhages (Loria et al., 2013) and even severely thrombocytopenic mice only develop local hemorrhages at sites of inflammation (Iannacone et al., 2008), which might explain the cardiac specificity of hemorrhages in Resiquimod-treated CFN mice. Systemic tissue damage incurred during this acute inflammatory phase might then trigger the subsequent chronic autoimmune response observed in this model. Both viral infections and tissue damage are known triggers of autoimmunity (Getts et al., 2013).

**Fig. 5.**
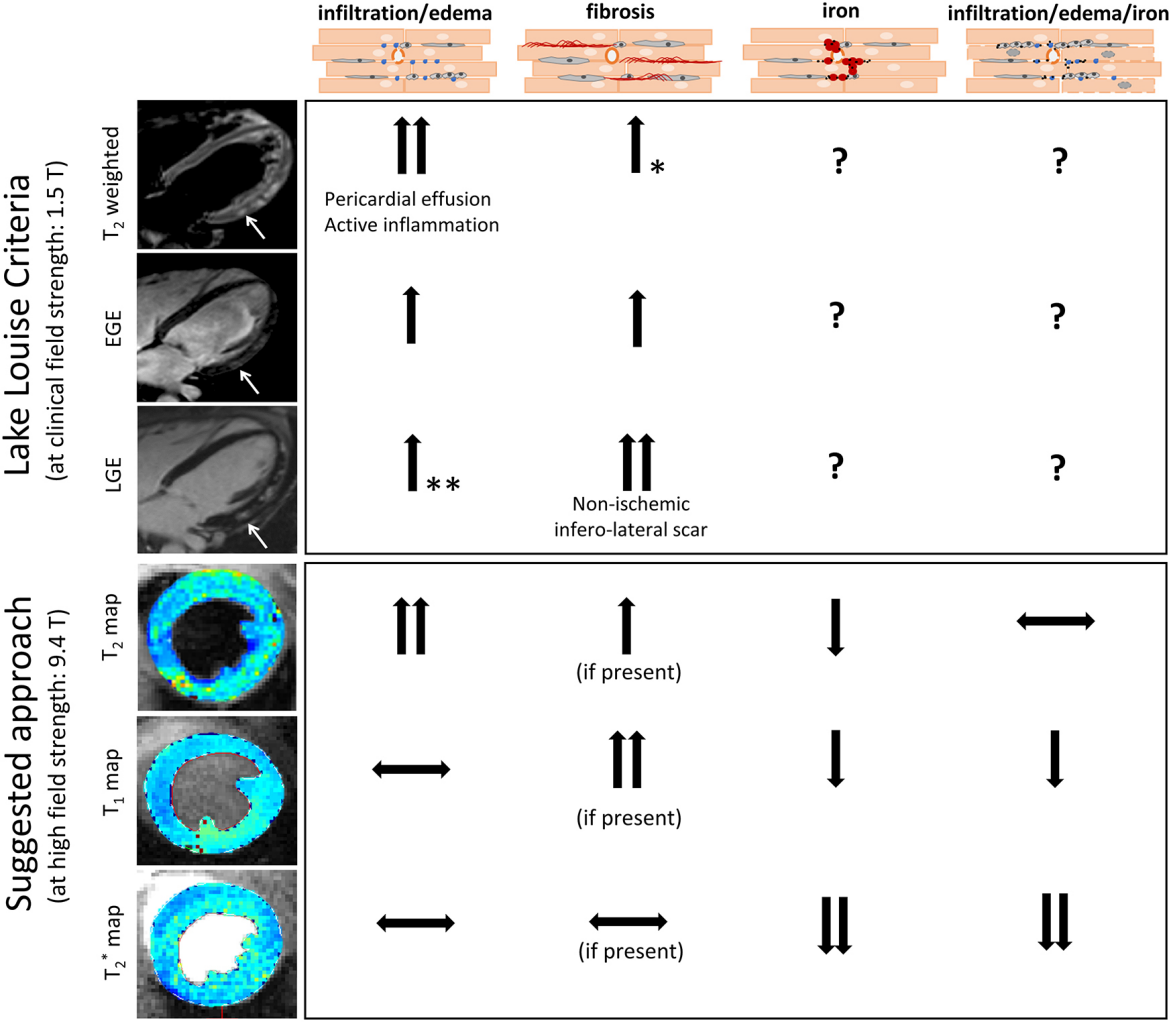
**Comparison of the CMR protocol presented in this study and the established diagnostic approach for myocarditis in human patients based on the Lake Louise criteria (LLC).** LLC-based measurements in human patients using clinical MRI field strengths. Edema leads to increased T_2_ weighted signal and increased T_2_ relaxation times, suggestive of acute inflammation and/or pericardial effusion. Hyperemia leads to increased early gadolinium enhancement (EGE) caused by leakage of gadolinium from capillaries. Necrosis or fibrosis can be identified by late gadolinium enhancement (LGE) because gadolinium crosses the damaged myocyte membrane (necrosis) or accumulates in the extended extracellular space (fibrosis). Example CMR images illustrate an example of increased edema (increased T_2_), capillary leakage (increased EGE) and nonischemic scar (positive LGE) at the inferolateral wall of a human myocarditis patient. *Fibrosis can also nonspecifically affect T_2_ values; however, T_2_ alone is not sufficient to diagnose fibrosis but needs to be combined with T_1_ or LGE measurements. **LGE can be enhanced in the presence of edema, but should not be used as a marker to identify myocardial edema, which should be done using T_2_ mapping. The approach proposed in this study and optimized on Resiquimod-treated mice with hemorrhagic myocarditis provides a contrast agent-free CMR protocol able to detect inflammatory damage and allows correct interpretation of pathology, despite potential interference from iron with the traditional T_1_ and T_2_ measures. In this model, edema increased and iron decreased T_2_ relaxation times, and values appeared unchanged in the presence of both edema and iron due to their opposing effects. Native T_1_ did not change in the presence of edema, and decreased in the presence of iron. T_1_ is expected to increase in the presence of fibrosis. T_2_* strongly decreases in the presence of iron and is not expected to change significantly in response to edema or fibrosis.

Diagnosis of human myocarditis often still relies on invasive procedures to obtain biopsy material for histology. In mouse models, the need to excise hearts for histology prevents longitudinal studies, which would significantly improve experimental design. Attempts to reduce the need for heart biopsies, as well as improve animal welfare and experimental design in preclinical studies, have resulted in significant advances in CMR imaging, which is now considered the clinical gold standard for non-invasive detection of myocarditis. Changes in T_1_ and T_2_ relaxation times are commonly used to detect parameters of inflammatory damage in the heart, including edema, immune cell infiltration and fibrosis. Fig. 5 illustrates how pathological changes in tissues affect CMR parameters measured as part of the Lake Louise criteria (LLC), which represent the first attempt to define a non-invasive diagnostic framework for myocarditis (Friedrich et al., 2009). The LLC protocol includes (1) T_2_ weighted images to detect myocardial edema by measuring the myocardial T_2_ intensity change normalized to skeletal muscle (T_2_-STIR), (2) early gadolinium enhancement (EGE) to detect reactive hyperemia and (3) late gadolinium enhancement (LGE) to assess tissue injury and/or fibrotic remodeling. Considering the possibility of hemorrhages, the approach taken in the present study includes the measurement of iron deposition through T_2_^*^ myocardial mapping. Because of its high sensitivity to paramagnetic iron, T_2_^*^ mapping is performed routinely in patients with suspected cardiac iron overload (Anderson et al., 2001) and β-thalassemia (Argyropoulou and Astrakas, 2007). A CMR imaging approach for detection of myocardial iron in a mouse model of β-thalassemia has also been reported recently (Jackson et al., 2017). In these previous studies, the LV free wall was excluded because of large magnetic susceptibility artefacts caused by proximity to the air-filled lung. Our approach reduced this susceptibility-induced fast T_2_^*^ decay by applying a UTE readout instead of the conventional gradient-echo readout (Robson et al., 2003), providing artifact-free parametric maps. We demonstrate its feasibility for detecting diffuse inflammatory infiltration and edema in mice with acute cardiac inflammation and identify the presence of interstitial iron as a result of hemorrhage, which is paralleled by a significant reduction in T_1_ and T_2_ relaxation times. Based on the above, we identified two main CMR phenotypes in hearts affected by inflammation and hemorrhage: (1) increased T_2_ values with normal T_1_, T_2_^*^, detecting areas of infiltration and/or edema with normal levels of iron in the tissue, suggestive of an acute ongoing inflammatory process as seen in classical myocarditis and (2) low T_2_^*^, T_1_, T_2_ values, detecting areas of infiltration and/or edema with increased levels of iron in the tissue, suggestive of inflammatory changes including vascular damage, red blood cell extravasation and tissue iron deposition. This may need to be taken into consideration when designing myocarditis imaging protocols to avoid false negative results.

In summary, we show that chronic anti-heart autoimmunity in the Resiquimod model of SLE follows acute thrombocytopenia and hemorrhagic myocarditis, and provide a thorough comparison of *in vivo* CMR parameter measurements and the underlying heart histopathology. The CMR protocol established in this study allows for detailed non-invasive characterization of diffuse myocardial inflammatory damage and controls for potential hemorrhage and iron deposition. The ability to discriminate different processes of tissue damage non-invasively by using specific MRI indices allows insight into the natural history of disease and improves our understanding of the potentially subclinical course of cardiac involvement in systemic inflammation, supporting the prognostic and diagnostic potential of this approach.

## MATERIALS AND METHODS

### Mice and *in vivo* treatment

All mouse procedures were approved by the Imperial College Governance Board for Animal Research and in accordance with the UK Home Office Animals (Scientific Procedures) Act 1986 and Directive 2010/63/EU of the European Parliament on the protection of animals used for scientific purposes. Experimental mice were 10- to 12-week-old male and female littermates. They were housed in individually ventilated cages in temperature-controlled facilities on a 12 h light/dark cycle on standard diet. The mouse line used in these studies was derived from a triple cross between C57BL/6J, FVB/NJ and NOD/ShiLtJ parental lines (CFN line) and displays high sensitivity to treatment with TLR-7 agonist Resiquimod (R848, Sigma-Aldrich, Dorset, UK) (Hasham et al., 2017). Treatment with Resiquimod was performed by topical application (100 μg/30 μl per 30 g body weight in 1:3 ethanol:acetone) to the ear three times a week, as previously described (Hasham et al., 2017).

### CMR

Mice were anesthetized and maintained under inhalation anesthesia via a nose cone (2% isoflurane in medical oxygen). Respiration, ECG and body temperature were continuously monitored (1030-MR, SA Instruments, Stony Brook, NY, USA) through the CMR scans. Three ECG leads (SA Instruments) were placed subcutaneously on the left and right sides of the thorax and on the right back leg; animals were positioned prone in a dedicated mouse bed. Body temperature was maintained at 36.5-37°C by a circulating warm water heat mat.

All CMR scans were performed on a preclinical 9.4T scanner (94/20 USR Bruker BioSpec; Bruker Biospin, Ettlingen, Germany) housed at the Biological Imaging Centre, Imperial College London using an 86 mm inner-diameter volume transmit quadrature coil combined with an actively decoupled mouse heart array receiver. Data were acquired with Paravision 6.0.1 (Bruker, BioSpin).

For localization of the heart, low-resolution ECG and respiratory triggered gradient-echo scout scans were acquired in axial, sagittal and coronal orientations followed by pseudo two- and four-chamber views. This allowed reproducible planning of imaging slices in the true short axis orientation at three specific locations in the left ventricle (basal, mid and apical). A multiparametric MR imaging approach was adopted and included mapping of T_1_, T_2_ and T_2_^*^ relaxation times to identify and characterize quantitatively the markers of inflammatory tissue injury observed in the Resiquimod-treated mice. For welfare reasons, because of the high susceptibility of Resiquimod-treated mice to acute bleeding, no contrast agent-based protocols were performed in this study.

#### T_1_ mapping

T_1_ mapping was performed (day 0, *n*=12; 2.5 weeks, *n*=9) using a gradient echo-based look-locker inversion recovery sequence with 20 inversion times (TI) that followed an adiabatic global inversion time. All inversions were R-wave triggered to allow images to be acquired at the same part of the cardiac cycle (end diastole), with the TI points restricted to multiples of the R–R interval (∼100 ms). Additional acquisition parameters were inversion repetition time of 5 s (to allow full relaxation between inversions), TR 4.5 ms, TE 2.1 ms, flip angle 7°, slice thickness 1 mm, field of view (22×22) mm^2^ and spatial resolution (164×164) μm^2^, with three slices acquired sequentially in ∼14 min. The inversion points acquired during respiratory motion were manually removed. The T_1_ curve fitting was subsequently performed on a pixelwise basis on the remaining measured inversion times using a nonlinear least square three-parameter fit, which accounted for Look–Locker correction. Data analysis was performed in Segment version 2.0 (Segment, Medviso) (Bidhult et al., 2016a,b).

#### T_2_ mapping

T_2_ mapping was performed (day 0, *n*=10; 2.5 weeks, *n*=10) using a multi-echo spin-echo fat-suppressed sequence with five echo times acquired at the same part of the cardiac cycle (proximal to end systole). A pair of flow saturation bands surrounding each imaging slice was placed upstream of the blood flow to minimize misleading signal dropouts caused by flow artefacts. Additional acquisition parameters were TR/TE 3500/2.74, 5.49, 8.23, 10.98 and 13.72 ms; echo spacing 2.74 ms; slice thickness 1 mm; field of view (17×19) mm^2^; spatial resolution (147×167) μm^2^ and GRAPPA acceleration factor 1.65, with three slices acquired sequentially in a total scan time of ∼12 min. A two-parameter pixelwise T_2_ fit was carried out assuming a mono-exponential signal decay. Myocardial segmentation excluded the regions of bright signal caused by the stagnant subendocardial blood occasionally observed on T_2_ maps (Giri et al., 2009). Data analysis was performed using Segment 2.0 (Segment, Medviso).

#### T_2_^*^ mapping

A multi-echo double cardiac and respiratory triggered two-dimensional radial UTE sequence was used for T_2_^*^ mapping (day 0, *n*=5; 2.5 weeks, *n*=3). For this, the sequence was performed repeatedly with various TEs (0.37, 1.5, 2.2 and 3 ms) with a fixed scale of receiver gain. The other imaging parameters were TR 5.6 ms, flip angle 12°, FOV (20×20) mm^2^, in-plane spatial resolution (164×164) µm^2^, slice thickness 1 mm, bandwidth 250 kHz, projection number 352 and a scan time of 3.5 min for each individual echo image. Precise k-space trajectory measurement is crucial for obtaining good quality images when radial encoding is employed (Zhang et al., 1998). Consequently, in this study, the radial trajectories were acquired *in vivo* from the signal of off-centered spins measured while playing out the gradient shapes in *X*, *Y* and *Z* directions, and measuring the phase difference of the retrieved signal (Zhang et al., 1998). To ensure that the measured trajectory was identical to the imaging gradients used, the ADC delay and the ramp-up shape of the readout gradient were included in the trajectory calibration. To improve accuracy of the *in vivo* trajectory measurement, we used 12 averages and ECG triggering. A pixelwise T_2_^*^ mono-exponential fit was performed on the multiple echo T_2_^*^w-UTE images using Segment 2.0 (Segment, Medviso) (BidhulT et al., 2016a,b).

#### CMR data analysis

Regional tissue characterization was performed on the T_1_, T_2_ and T_2_^*^ maps after partitioning the left ventricle wall based on the 16-segmentation model, as per recommendations by the American Heart Association (six segments for basal and mid level, four segments for the apex) (Cerqueira et al., 2002). Epicardial and endocardial outlines were traced manually to define the myocardial wall of each animal (Tufvesson et al., 2015). Particular care was taken to avoid signal contamination from the LV blood pool by excluding the innermost 5% of the myocardial wall. A layered pattern of signal dropout was observed surrounding primarily the subepicardial regions of the intraventricular septum in most of the mice. To account for this, each of the six cardiac segments of the midventricular slice was divided into two equal sections (subepicardial and subendocardial, average pixel number per segment of 36±5), resulting in 12 segments per slice (Fig. 2). Signals of each of the 12 segments were normally distributed, therefore T_1_, T_2_ and T_2_^*^ average values were computed and correlated with histology as described below. Correlation of MRI to histology data was performed using midline cross-section values. Voxel intensity histograms of the T_1_ and T_2_ maps were extracted over the full myocardial thickness of the midventricular anterior septum and anterior lateral walls of both Resiquimod-treated and untreated mice. Histograms of T_2_^*^ maps were then compared with their respective T_1_ and T_2_ histograms to further evaluate their interdependence and to better characterize their specific distribution pattern in the presence of inflammatory tissue injury and myocardial bleeding. Histograms were reconstructed using 20 bins, mean or median, as appropriate, which were measured and used for further analysis. To better visualize the specific features of signal distribution of each CMR mapping technique, smoothing was performed by averaging two values on each side and using a second-order polynomial smoothing (Savitzky and Golay, 1964).

### Macroscopic observations and scoring

Hearts were perfused and excised. Macroscopically visible hemorrhagic lesions were scored on a scale from 0 to 4: (0) no visible lesions, (1) lesions cover <10% of heart surface, (2) lesions cover 10-30% of heart surface, (3) lesions cover 30–50% of heart surface and (4) lesions cover >50% of heart surface.

### Histology and scoring of damage parameters

Hearts of treated and untreated mice were excised after *in situ* perfusion with ice-cold phosphate buffered saline (PBS; Sigma-Aldrich) through the apex of the left ventricle of the heart to clear blood from heart chambers and blood vessels. Hearts were then fixed in 4% formaldehyde overnight, dehydrated in an increasing gradient of ethanol and embedded in paraffin. Sections of 5 µm were cut, de-waxed and then rehydrated in an ethanol gradient. Sections were stained with H&E, Picrosirius Red and Perls Prussian Blue. All reagents were purchased from Sigma-Aldrich. Semiquantitative scoring was performed as established previously (Hasham et al., 2017). H&E-stained sections were used to analyze extravasation of red blood cells and mononuclear cell infiltration. Picrosirius Red staining was used to analyze and score fibrosis. Perls Prussian Blue staining was used to analyze and score cardiac iron deposition. Individual parameters were scored on a scale from 0 to 3: (0) none, (1) mild, (2) moderate and (3) severe. Scores were obtained from 12 segments along the myocardium at 100× magnification on four midline cross-sections per animal by a blinded researcher. Images were captured using a LMD7000 microscope (Leica Microsystems, Milton Keynes, UK) and processed using the public domain software ImageJ (NIH; http://rsb.info.nih.gov), (Schneider et al., 2012).

### Hematological analysis

Blood was collected into 129 mM trisodium citrate and platelet counts were determined immediately by flow cytometry using a rat antibody recognizing mouse platelet GPIbβ (Emfret Analytics, Eibelstadt, Germany) and calibrated beads (Saxon Europe, Kelso, UK) according to manufacturer's instructions. Samples were analyzed using a FACScalibur flow cytometer. Hemoglobin content in blood samples was determined by the cyan-methemoglobin method using Drabkin's reagent and bovine hemoglobin as a standard (both from Sigma-Aldrich). Blood or hemoglobin standards were diluted in 200 µl Drabkin/Brij L23 solution, incubated at room temperature for 15 min and absorbance read at 540 nm. Hemoglobin content in mouse blood samples were extrapolated from a standard curve of bovine hemoglobin by linear regression using GraphPad Prism (v7).

### Experimental design and statistical analysis

Calculations of animal numbers and sample sizes were performed using G*Power 3.1 (Faul et al., 2007), available at http://www.gpower.hhu.de/, and reflect effect sizes obtained in previous experiments with comparable readouts. Scoring of histopathology was performed by a blinded researcher. Statistical analyzes were performed using SPSS or GraphPad Prism. Data are presented as mean±s.e.m. The Mann–Whitney test was used for scoring data, and one- or two-tailed unpaired or paired Student's *t*-tests were performed as appropriate for parametric data.

## Supplementary Material

Supplementary information
