# Supplementary material for: Characterization of acute TLR-7 agonist-induced hemorrhagic myocarditis in mice by multiparametric quantitative cardiac magnetic resonance imaging
Source: Dis Model Mech. 2019 Aug 16;12(8):dmm040725. doi: 10.1242/dmm.040725 (PMC6737951; doi:10.1242/dmm.040725)
Supplement: Supplementary information [file dmm-12-040725-s1.pdf]

Supplementary Figure 1

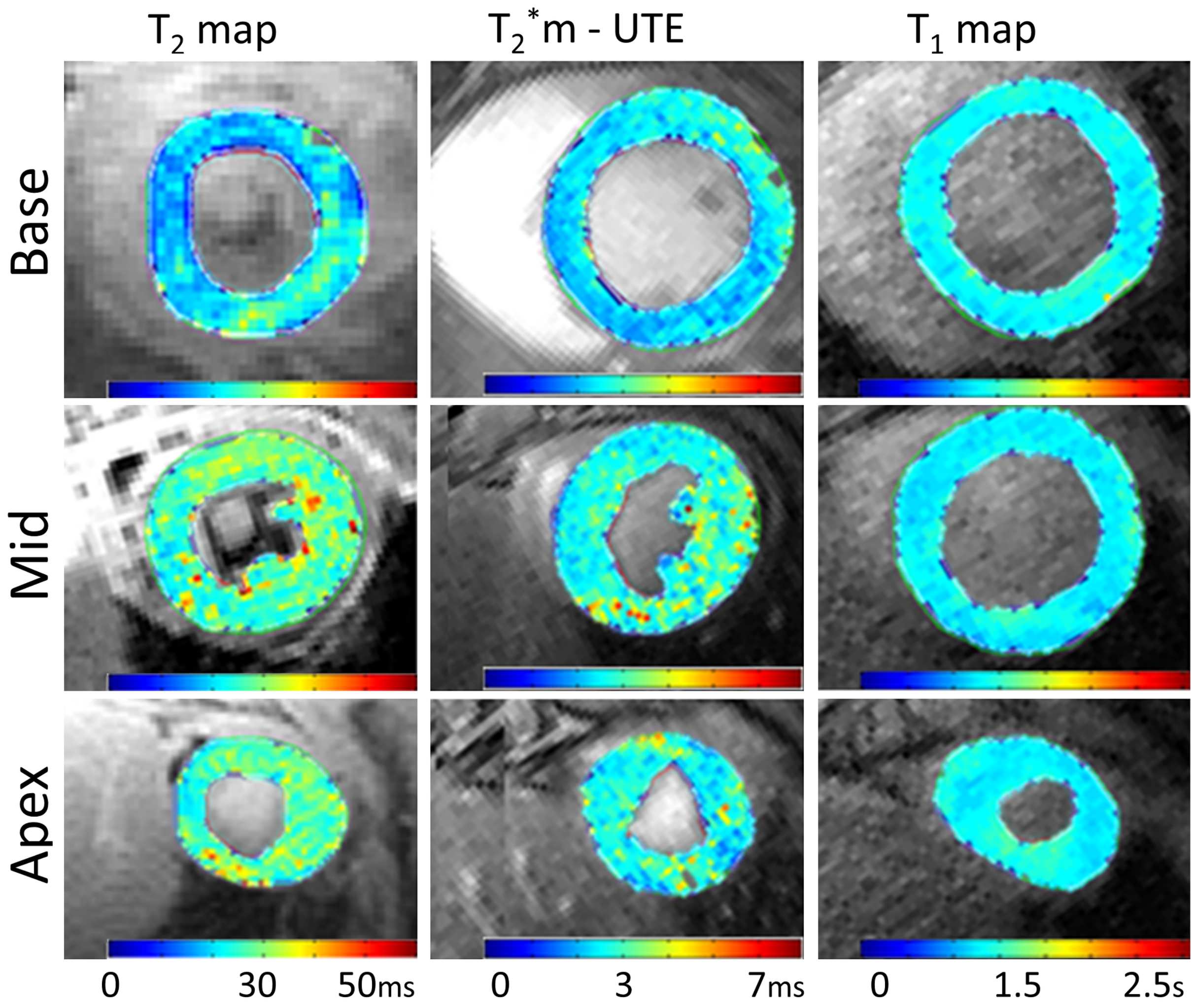


| MRI Methods | $T_2$map (MSSE) Base, Mid, Apex | $T_2^*$ map (UTE) Base, Mid, Apex | Native $T_1$ map (Look-Locker) Base, Mid, Apex |
|---|---|---|---|
| **Diffuse myocardial involvement** | Oedema/ Inflammation | Bleeding/Iron | Diffuse fibrosis |

*~ 4 min/slice* *~ 14 min/slice* *~ 4 min 30s /slice*

*No contrast agent*

**Figure S1:** Improved multi-parametric approach to assess tissue damage by $T_1$, $T_2$ and $T_2^*$ mapping. Representative $T_1$, $T_2^*$ and $T_2$ maps acquired at base, midsection and apex of healthy mice showing the fesibility of this approach.

Supplementary Figure 2

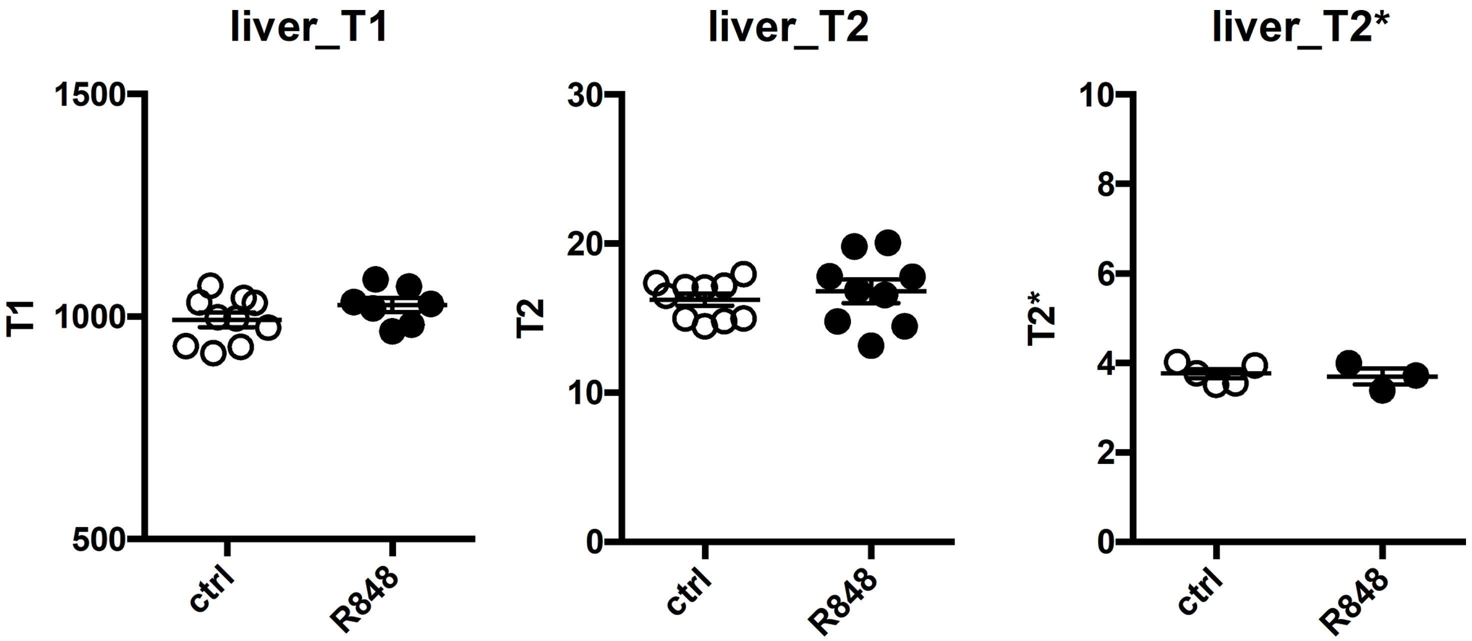


**Figure S2:** MRI mapping parameters in the liver appear largely normal. Potential iron deposition and inflammatory damage to the liver in Resiquimod treated mice was assessed by $T_1$, $T_2$ and $T_2^*$ measurements in MRI.
